# Supplementary material for: Induction of Robust and Specific Humoral and Cellular Immune Responses by Bovine Viral Diarrhea Virus Virus-Like Particles (BVDV-VLPs) Engineered with Baculovirus Expression Vector System
Source: Vaccines (Basel). 2021 Apr 6;9(4):350. doi: 10.3390/vaccines9040350 (PMC8067437; doi:10.3390/vaccines9040350)
Supplement: Supplementary file 1 [file vaccines-09-00350-s001.pdf]

Supporting information

Induction of Robust and Specific Humoral and Cellular Immune Responses by Bovine Viral Diarrhea Virus Virus-Like Particles (BVDV-VLPs) Engineered with Baculovirus Expression Vector System

Zhanhui Wang 1, Mengyao Liu 1, Haoran Zhao 1, Pengpeng Wang 1, Wenge Ma 1, Yunke Zhang 1, Wenxue Wu 1* and Chen Peng 1*

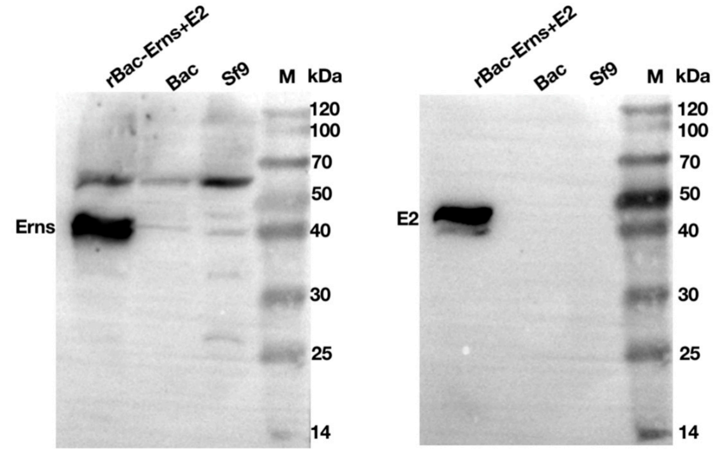

Figure S1. Expression of E^{rns} and E2 proteins in Sf9 Cells. Western blot analysis of E^{rns} and E2 expression in Sf9 cells, cell lysates from Sf9 cells infected with rBac-E^{rns}+E2 were subjected to western blot analyses and E^{rns} and E2 were detected by E^{rns} pAb and E2 mAb 348, respectively. Cell lysates from Sf9 cells uninfected or infected with Bac vector were loaded as controls, numbers on the right indicate protein molecular weight in kDa.

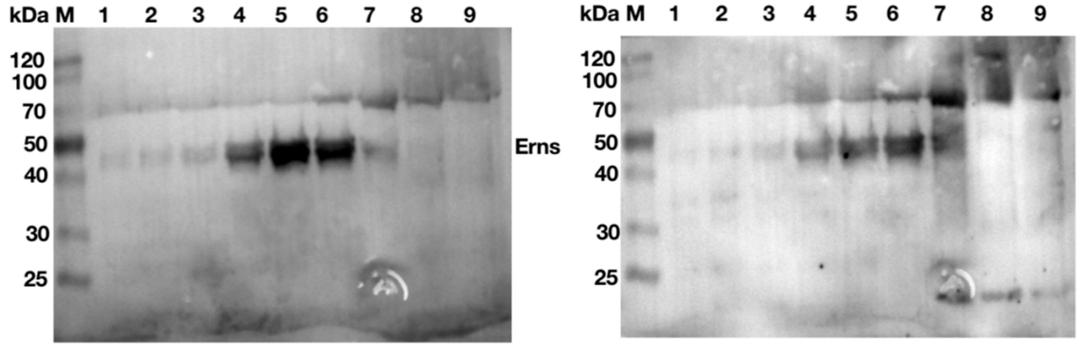

Figure S2. Purification of VLPs by sucrose density centrifugation. Western blot analysis of fractions of 1-9 with E^{rns} pAb and E2 mAb 348, numbers on the left indicate protein molecular weight in kDa.

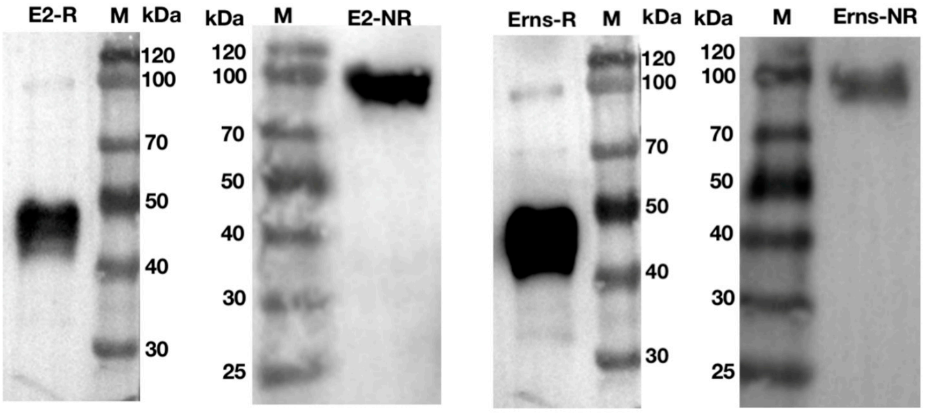

Figure S3. Homodimerization of E^{rns} or E2 proteins. The VLPs were separated by SDS-PAGE under R or NR conditions followed by Western blotting with E^{rns} pAb and E2 mAb 348, on the left/right protein ladder, the molecular weight in kDa is given.
